# Polydatin alleviated radiation‐induced lung injury through activation of Sirt3 and inhibition of epithelial–mesenchymal transition

**DOI:** 10.1111/jcmm.13230

**Published:** 2017-06-13

**Authors:** Kun Cao, Xiao Lei, Hu Liu, Hainan Zhao, Jiaming Guo, Yuanyuan Chen, Yang Xu, Ying Cheng, Cong Liu, Jianguo Cui, Bailong Li, Jianming Cai, Fu Gao, Yanyong Yang

**Affiliations:** ^1^ Department of Radiation Medicine Faculty of Naval Medicine Second Military Medical University Shanghai China

**Keywords:** polydatin (PD), radiation‐induced lung injury, free radicals, epithelial–mesenchymal transition (EMT)

## Abstract

Radiation‐induced lung injury (RILI) is one of the most common and fatal complications of thoracic radiotherapy. It is characterized with two main features including early radiation pneumonitis and fibrosis in later phase. This study was to investigate the potential radioprotective effects of polydatin (PD), which was shown to exert anti‐inflammation and anti‐oxidative capacities in other diseases. In this study, we demonstrated that PD‐mitigated acute inflammation and late fibrosis caused by irradiation. PD treatment inhibited TGF‐β1‐Smad3 signalling pathway and epithelial–mesenchymal transition. Moreover, radiation‐induced imbalance of Th1/Th2 was also alleviated by PD treatment. Besides its free radical scavenging capacity, PD induced a huge increase of Sirt3 in culture cells and lung tissues. The level of Nrf2 and PGC1α in lung tissues was also elevated. In conclusion, our data showed that PD attenuated radiation‐induced lung injury through inhibiting epithelial–mesenchymal transition and increased the expression of Sirt3, suggesting PD as a novel potential radioprotector for RILI.

## Introduction

Radiation‐induced lung injury (RILI) is a most common and serious complication in radiotherapy for thoracic tumours 1. The development of RILI is mainly divided into an early‐phase characterized as radiation pneumonitis, and a later phase of chronic pulmonary fibrosis. Pneumonitis phase occurs within 3 months after high doses of irradiation (more than 8 Gy), while fibrosis phase presents months to years later. The main clinical symptoms of RILI were inflammatory infiltration of interstitial fluid, progressive dyspnea, deterioration of pulmonary function and eventually leading to respiratory failure 2. Moreover, it also limits the further application of fractionated radiotherapy. Although steroids had been used in the treatment of RILI, severe side effects remain a huge challenge 3, 4, 5. It is urgently required to develop novel safe and effective therapeutic strategies for RILI.

Ionizing radiation (IR) results in cellular damage by direct deposit of energy and indirect oxidative damage by the free radicals produced from radiolysis of water. Injuries and apoptosis of alveolar and vascular cells lead to loss of junctional integrity, increased permeability and physiological dysfunction 6. IR also induces burst of inflammatory cytokines and chemokines, which causes infiltration of inflammatory cells such as macrophages and neutrophils 7, 8. TGFβ signalling pathway has been shown to play an important role in RILI 9, 10. It was also reported that radiation induced a lasting Th2‐like immune response which caused immune imbalance 11, 12. However, the exact mechanism of RILI remains unclear.

Polydatin (PD) is a natural presuror of resveratrol, which is extracted from toot of polygonum cuspidatum and exhibits antioxidant, anti‐tumour and anti‐inflammatory properties. In the resveratrol, there are three hydroxyl group in 3, 4, 5 position of stilbene scaffold, while in polydatin the hydroxyl group is replaced with a glucopyranoside ring 13. This substitution leads to conformation changes, which results in changes in its biological properties. Compared to resveratrol, PD is more efficiently absorbed, more resistant to enzymatic oxidation and even soluble in hot water. Different from resveratrol, PD enters cells *via* active mechanism using glucose carriers 14. These properties provide PD better bioavailability than resveratrol. PD retains powerful free radical scavenging property, and also exhibits anti‐oxidant functions 15, 16, 17. PD was also shown to exert cardioprotective effects against ischemia/reperfusion injury 18, 19. It was also reported that PD protected burn‐induced lung injury and lipopolysaccharide (LPS)‐induced lung injury 20, 21. We hypothesized that whether it could protect radiation‐induced lung injury, which shows great potential in thoracic radiotherapy. In addition, low toxicity of PD shows good biological safety. In this study, we demonstrated that PD effectively suppressed radiation pneumonitis and chronic pulmonary fibrosis through inhibiting EMT and activating of Sirt3‐related signalling pathways.

## Materials and methods

### Animals and PD treatments

All the protocols were approved by the Animal Ethics Committee of Second Military Medical University, China, in accordance with the Guide for Care and Use of Laboratory Animals published by the US National Institute of Health (publication no. 96‐01). Female C57BL/6 mice, 8 weeks old, were obtained from the Experimental Animal Center of Chinese Academy of Sciences, Shanghai, China. Mice were randomly divided into four groups: group 1, non‐irradiated + saline control (*n* = 30); group 2, irradiation + saline (*n* = 30); group 3, irradiation + Polydatin (*n* = 30) and group 4, irradiation + WR2721 (*n* = 30). Mice were housed six per cage and kept under standard laboratory conditions (22 ± 2°C, 55 ± 10% humidity and 12‐12 hr/light–dark cycle), during which sterilized food and water were supplied *ad libitum*. Polydatin was obtained from Sigma‐Aldrich Co. (America). Polydatin was given by intraperitoneal injection 3 days before irradiation at the dose of 100 mg/kg/day and maintained for 4 weeks after irradiation. WR2721 (100 mg/kg/day) and saline were administered by intraperitoneal injection at the same time in different groups.

### Cell culture and PD treatments

Human bronchial epithelial cell line BEAS‐2B (American Type Culture Collection) was maintained in RMPI 1640 medium (10% foetal bovine serum) at 37°C in a 5% CO_2_ humidified chamber. BEAS‐2B cells were pre‐treated with or without PD at 1 hr before irradiation and further cultured for another 24 hrs then switched to the normal RMPI1640 medium. For knock‐down of Sirt3, a Sirt3 siRNA or a negative control was transfected into BEAS‐2B cells at 48 hrs before irradiation, and then cells were subjected to PD treatment and irradiation.

### Irradiation

^60^Co source in the Irradiation Center (Faculty of Naval Medicine, Second Military Medical University, China) was used for irradiation. After anesthetization with 10% chloral hydrate (350 mg/kg), mice were put in a holder designed to immobilize anaesthetized mice so that only the whole thorax was exposed to the beam. All radiated animals received a single dose of 15 Gy with a dose rate of 1 Gy/min and were monitored up to 16‐week post‐irradiation. BEAS‐2B was irradiated with 8 Gy at a dose rate of 2 Gy/min.

### Sample collection and initial processing

Six mice at each time‐point (1, 2, 4, 8 and 16 weeks post‐irradiation) from different groups were killed by cervical dislocation after anesthetization. After measuring body weight, cardiac puncture was performed to obtain about 1 ml blood. The blood was allowed to clot at room temperature for 2 hr and then centrifuged at 3000 g 4°C for 15 min. The serum was collected and stored at −80°C for latter analysis by enzyme‐linked immunosorbent assay (ELISA). Wet weight of lung in each group was recorded for each mouse, the lower part of left lung was fixed with 4% paraformaldehyde for at least 48 hrs before histological and immunofluorescence analysis. The rest of the lungs were divided into four parts and kept at −80°C for determination of lipid peroxides (MDA), SOD activities, hydroxyproline and Western blot analyses, respectively.

### Lung coefficient

The body weight and lung wet weight were measured at 1, 2, 4, 8 and 16 weeks post‐irradiation. The ratio of the lung wet weight (mg) to body weight (g) was used as lung coefficient.

### Histopathology and immunohistochemistry

Lung tissues was used for terminal transferase‐mediated dUTP nick end labelling (TUNEL) and stained with H&E and Masson's trichrome, antibodies for TGF‐β1 (1:200; Cell Signaling Tech., MA, USA), p‐Smad3 (1:200; Cell Signaling Tech.), Snail (1:200; Cell Signaling Tech.), Sirt3 (1:200; Cell Signaling Tech.), Nrf2 (1:200; Cell Signaling Tech.) and PGC1α (1:100; Cell Signaling Tech.). Five fields per section at ×200 magnifications were randomly selected per mouse, and two blinded pathologists independently examined 30 fields per group using Nikon DS‐Fi1‐U2 microscope (Nikon, Tokyo, Japan). The mean score of all fields examined was taken as the fibrosis score of each animal.

### Immunofluorescence staining

Immunofluorescence analysis was performed to measure the expression of Vimentin, E‐cadherin, α‐SMA in lung tissues. After deparaffinization, antigen retrieval and incubation with Rodent Block M, the sections were incubated with a mixture of anti‐Vimentin (1:500, Cell Signaling Tech.), anti‐E‐cadherin (1:500, Cell Signaling Tech.) and anti‐α‐SMA (1:500, Cell Signaling Tech.) antibodies at 4°C overnight. After washed with PBS, the sections were incubated with FITC‐conjugated goat anti‐mouse (Invitrogen, Carlsbad, CA, USA) an Texas Red‐conjugated anti‐rabbit secondary antibodies (Cell Signaling Tech.) at room temperature for 30 min. Nuclei were counterstained with DAPI, and the sections were analysed using a fluorescence microscope (Nikon Eclipse Ti‐SR, Nikon, Tokyo, Japan).

### Western blot analysis

Frozen lung tissue was homogenized in mammalian protein extraction reagent (M‐PER) to prepare a protein sample. Lysates of BEAS‐2B cell were prepared similarly. The lysates were mixed with 10% SDS‐PAGE then electrophoresis was performed on the same volume. After the electrophoresis, the protein was transferred to a nitrocellulose membrane (Amersham, Arlington Heights, IL, USA) then blocked with 5% milk for 1 hr at room temperature. The proteins were incubated with Snail (1:1000; Cell Signaling Tech.), E‐cadherin (1:1000; Cell Signaling Tech.), Vimentin (1:1000; Cell Signaling Tech.), α‐SMA (1:1000; Cell Signaling Tech.), β‐actin (1:1000; Cell Signaling Tech.), Sirt1 (1:1000; Cell Signaling Tech.), Sirt2 (1:1000; Cell Signaling Tech.), Sirt3 (1:1000; Cell Signaling Tech.), Sirt6 (1:1000; Cell Signaling Tech.) antibodies at 4°C overnight in a shaker incubator. After washing with TBS‐T, the membranes were incubated with anti‐rabbit IgG horseradish peroxidase conjugated antibody (1:5000; Cell Signaling Tech.) for 1 hr at room temperature. The protein bands were visualized using enhanced chemiluminescence with a Super Signal west pico kit (Bridgen Biological Technology, Shanghai, China). Films were scanned and analysed by densitometry using Syngene GeneGenius software (Syngene, Frederick, MD, USA).

### ELISA assays

Serum levels of TNF‐α, ET‐1, IL‐4, IL‐13, IFN‐γ, PGE2 and TGF‐β1 were determined using an ELISA kit according to the manufacturer's instructions. The OD value was determined at 450 nm using a DENLEY DRAGON Wellscan MK 3 (Thermo Fisher Scientific Inc. Waltham, MA, USA) and calculated at the linear portion of the curve.

### Measurement of lungs collagen content, MDA, SOD activities

The concentration of hydroxyproline was measured by a Hyp assay kit (Nanjing KeyGen Biotech., Nanjing, China) according to the manufacturer's instructions. Malondialdehyde (MDA) content and SOD activity in lung tissues were measured using commercialized chemical assay kits (Nanjing KeyGen Biotech.) according to the manufacturer's protocol. The total protein concentration was determined by a BCA (Bicinchoninic Acid) protein assay kit (Beyotime Institute of Biotechnology, Nantong, China).

### Electron spin resonance spectrometry

We used 1,1‐diphenyl‐2‐dinitrophenylhydrazine (DPPH; Labotec, Tokyo, Japan) anhydrous ethanol solution to prepare free radicals and detected electron spin resonance (ESR) signals *via* an ESR spectrometer (EMX‐8; Bruker BioSpin Corp, Berlin, Germany). We determined the scavenging capacity of PD for oxygen free radicals at different concentrations.

### Statistical analysis

Data were expressed at the means ± standard error of mean (SEM). Between group differences were tested using a one‐way ANOVA. Two‐group comparisons were performed using independent‐samples Student's *t*‐test. *P* < 0.05 was considered significant.

## Results

### Polydatin reduced hyperaemia and oedema in lung tissues after irradiation

**Figure 1:**
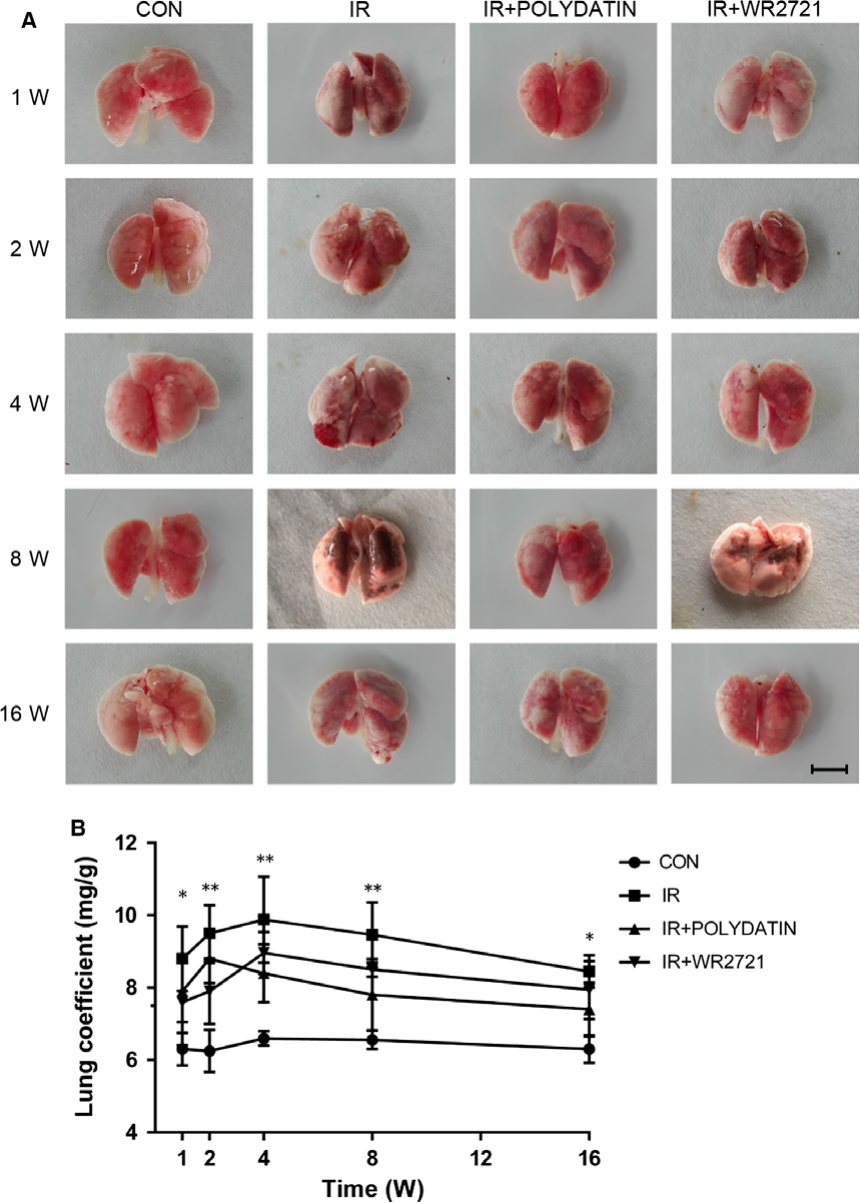
Polydatin reduced radiation‐caused hyperaemia oedema in pulmonary tissue. (**A**) Representative images of lung tissues in control group, single irradiation group, polydatin group and WR2721 group from 1 to 16 weeks. (**B**) A line graph of lung coefficient in different groups following local irradiation. Values are given as mean ± SEM (*n* = 30), *P < 0.05 and **P < 0.01 *versus* single radiation group.

In single radiation group, general view of lungs showed progressive bleeding points, congestion and oedema from 1 to 8 week post‐irradiation. And in PD treated group, the congestion and oedema of lung were obviously mitigated, and the area of haemorrhage was smaller than the single irradiation group. Moreover, general view of lung in PD group was better than that in WR2721 group (Fig. 1A). On the other hand, lung coefficient (lung weight/body weight) mainly reflects the degree of pulmonary oedema. Our data showed that lung coefficient increased and was significantly reduced by PD treatment (Fig. 1B).

### Polydatin mitigated structural damages and collagen deposit induced by irradiation

**Figure 2:**
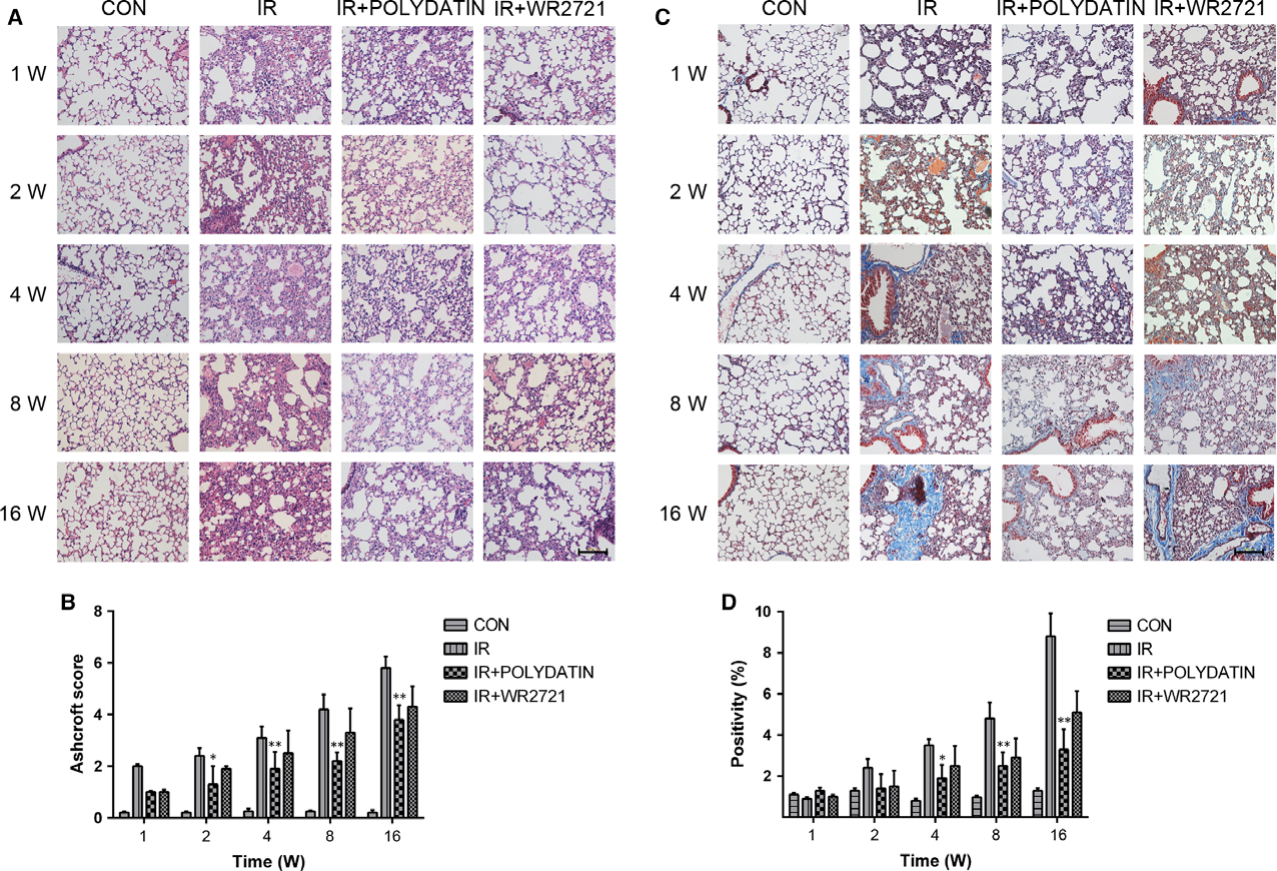
Polydatin mitigated radiation pneumonia and collagen deposition in lung tissues. Representative images of HE (**A**) and Masson staining (**C**) of lung tissue sections at 1–16 week post‐irradiation. (**B**) A bar graph of Ashcroft scoring of HE slides of lung tissues. (**D**) A quantification of collagen deposition area in slides from lung tissues. Values are given as mean ± SEM (*n* = 30), *P < 0.05 and **P < 0.01 *versus* single radiation group.

After 15 Gy local irradiation, alveolar septal thickening and inflammatory cells infiltration were observed in sections of lung tissues (Fig. 2A). Using a Masson's trichrome staining method, we found that radiation induced severe deposition of collagen, especially at 8 and 16 weeks (Fig. 2C). Compared with the single irradiation group, PD attenuated radiation‐induced inflammation in early stage and reduced collagen deposit in latter phase (Fig. 2A and C). Through quantifying analysis, we found that polydatin treatment group had a significant inhibitory effect on the occurrence of early radiation‐induced pneumonia and late radiation‐induced pulmonary fibrosis (Fig. 2C and D).

### Polydatin inhibited radiation‐induced epithelial–mesenchymal transition in lung tissues

**Figure 3:**
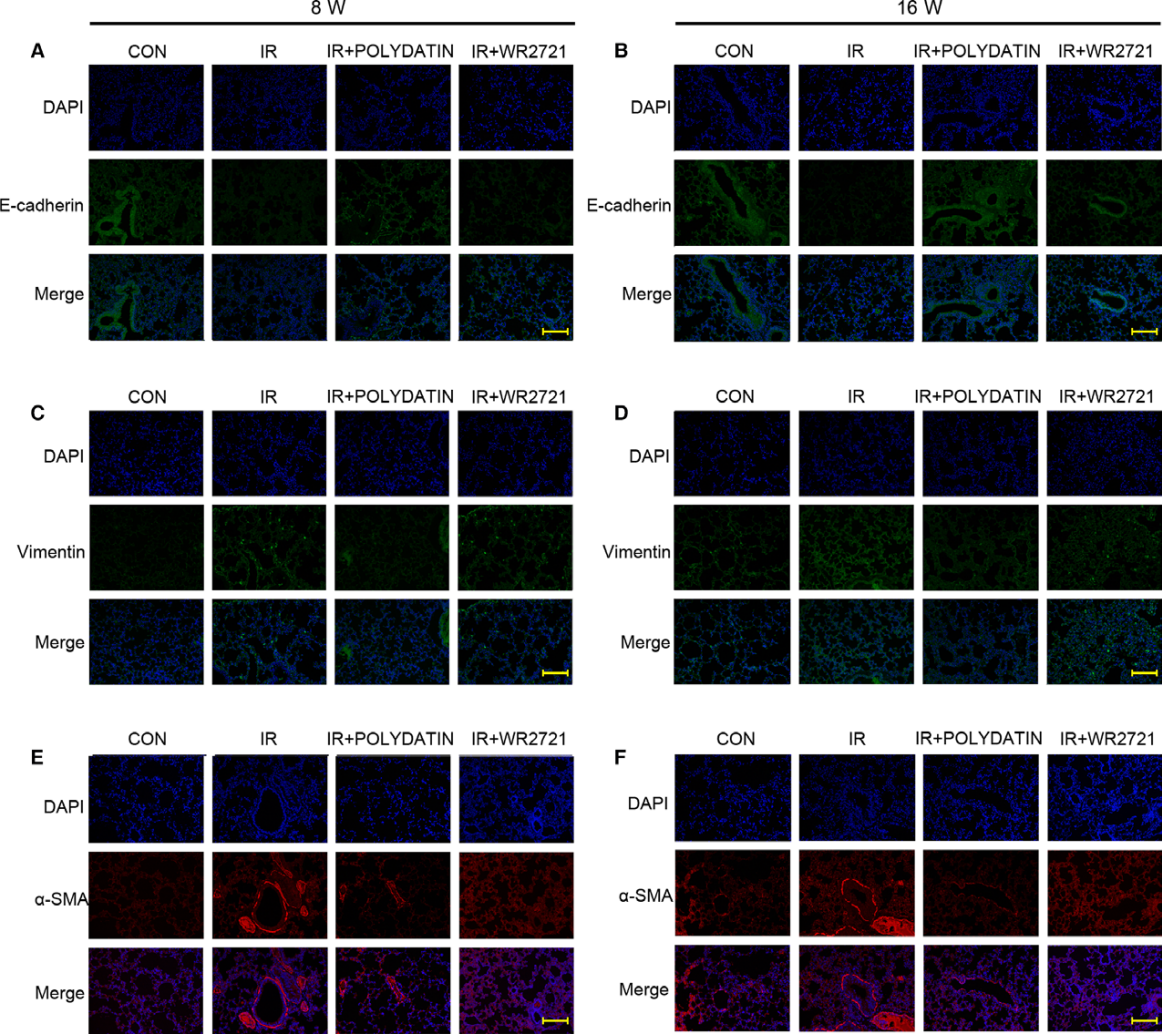
Polydatin inhibited radiation‐induced epithelial–mesenchymal transition. Representative images of EMT markers, E‐cadherin (**A**,** B**), Vimentin (**C**,** D**) and α‐SMA (**E**,** F**) IF staining in different groups at 8‐ and 16‐week post‐irradiation.

**Figure 4:**
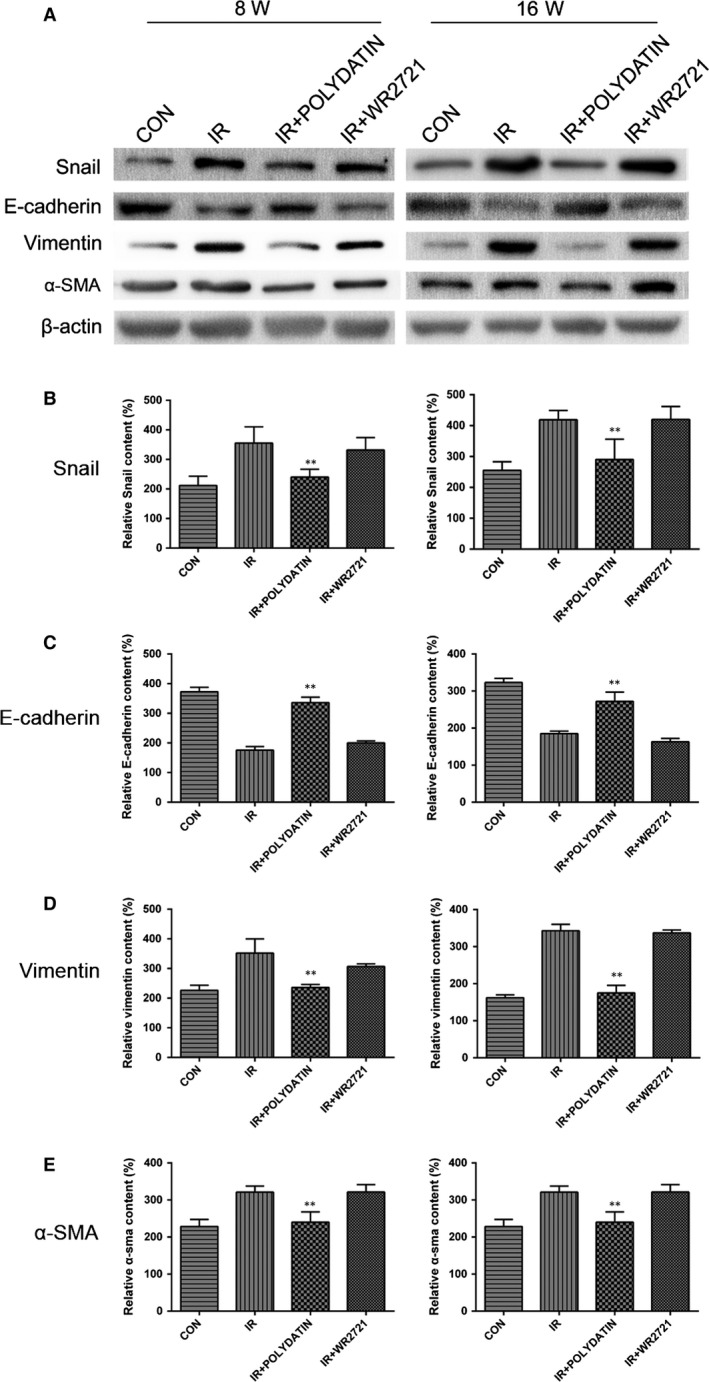
Polydatin reversed radiation‐induced EMT process evaluated by a Western blotting assay. (**A**) Western blot analysis of EMT markers in lung tissues from different groups. (**B**–**E**) Qualification of protein expression levels of E‐cadherin, Vimentin and α‐SMA in different groups. Values are given as mean ± SEM (*n* = 10), *P < 0.05 and **P < 0.01 *versus* single radiation group.

**Figure 5:**
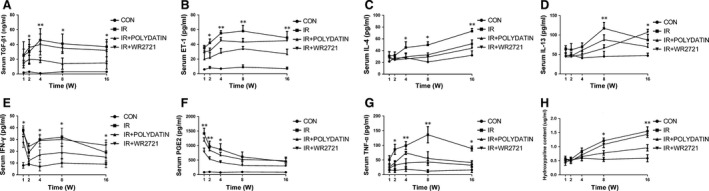
Polydatin regulated Th1/Th2‐related cytokines in acute lung injury. (**A**–**H**) Cytokines concentration in serum of irradiated mice with different treatments. Values are given as mean ± SEM (*n* = 10), *P < 0.05 and **P < 0.01 *versus* single radiation group.

Radiation‐induced epithelial–mesenchymal transition (EMT) is closely related to the development of pulmonary fibrosis. Immunofluorescence staining showed that at 8 and 16 week after irradiation, the epithelial marker E‐cadherin was progressive down‐regulated and the interstitial markers Vimentin and α‐SMA were significantly up‐regulated. In polydatin treatment group, the upregulation of interstitial markers Vimentin and α‐SMA and E‐cadherin decrease was significantly inhibited (Fig. 3A–F). However, the WR2721 treatment had no inhibitory effect on EMT process. Using a Western Blot assay, we confirmed that polydatin inhibited EMT process in irradiated lung tissues and in alveolar epithelial cell lines (Figs. 4, 8D–H). The content of hydroxyproline was also significantly inhibited by polydatin treatment (Fig. 5H).

### Polydatin suppressed inflammatory cytokines and TGF‐β‐Smad3 signalling pathway

**Figure 6:**
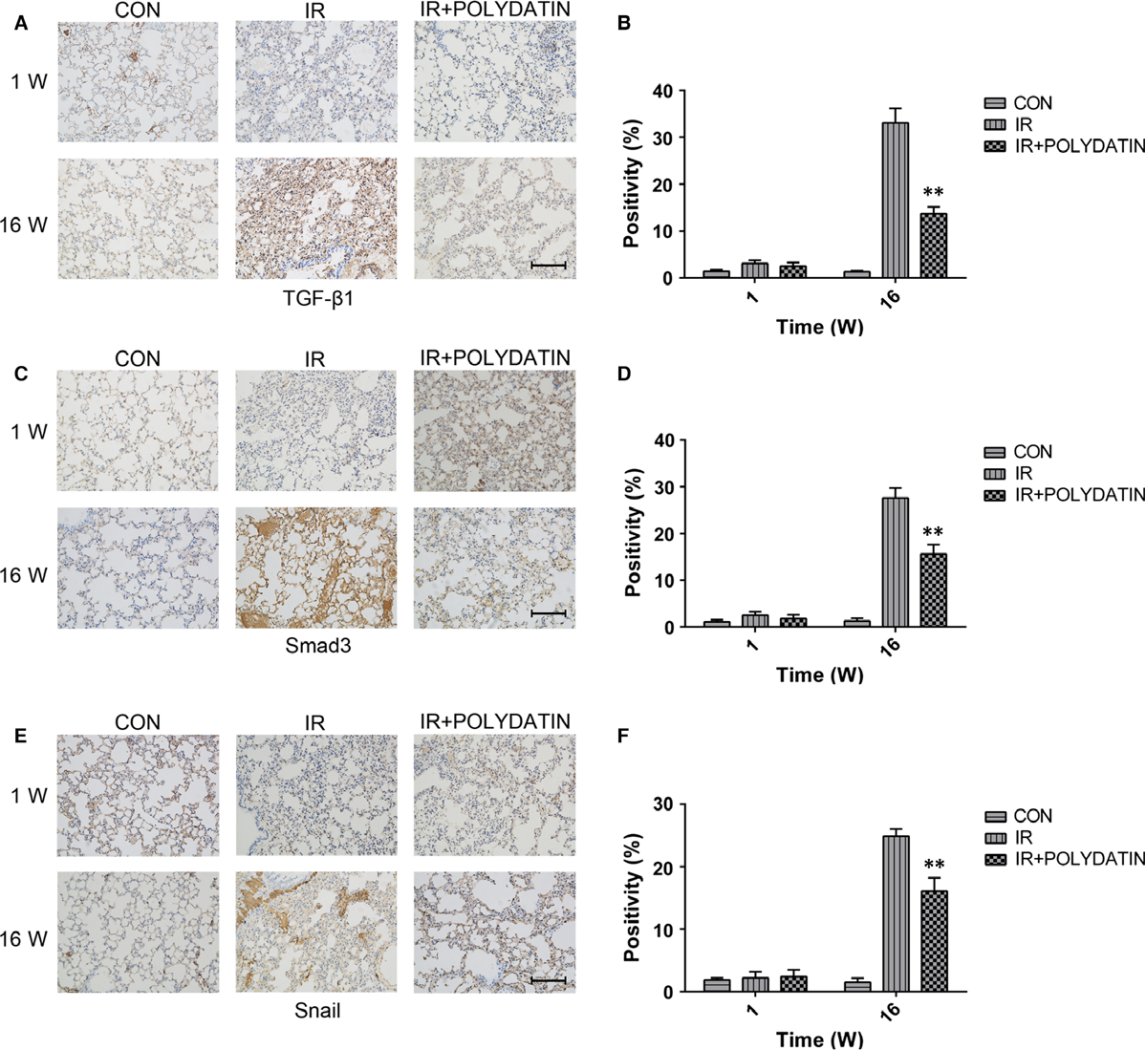
Polydatin reduced the expression levels of TGF‐β1, Smad3 and Snail. (**A**,** C**,** E**) Representative images of IHC staining of TGF‐β1, Smad3 and Snail in lung tissues from different groups at 1 and 16 week post‐irradiation. (**B**–**D**) Bar graphs showing quantification analysis of TGF‐β1, Smad3 and Snail‐positive cells in slide from lung tissues. Values are given as mean ± SEM (*n* = 10), *P < 0.05 and **P < 0.01 *versus* single radiation group.

Radiation induced significant increases in serum inflammatory cytokines (IL‐4, IL‐13, PGE2, TNF‐α) and TGF‐β1, which was significantly inhibited by polydatin treatment (Fig. 5). WR2721 also significantly reduced inflammatory cytokines, but no difference was found, compared with the polydatin group (Fig. 5). To check the expression of TGF‐β‐related proteins in lung tissues, we performed immunohistochemical staining of lung tissue in 1 and 16 week in mice, the results showed that there was no significant difference between different groups at 1 week after irradiation. While at 16 weeks after irradiation, TGF‐beta 1/Smad3/Snail pathway was up‐regulated in single radiation group, which was significantly inhibited by polydatin treatment (Fig. 6A–F).

### Polydatin scavenged free radicals, inhibited oxidative injury and cell death caused by irradiation

**Figure 7:**
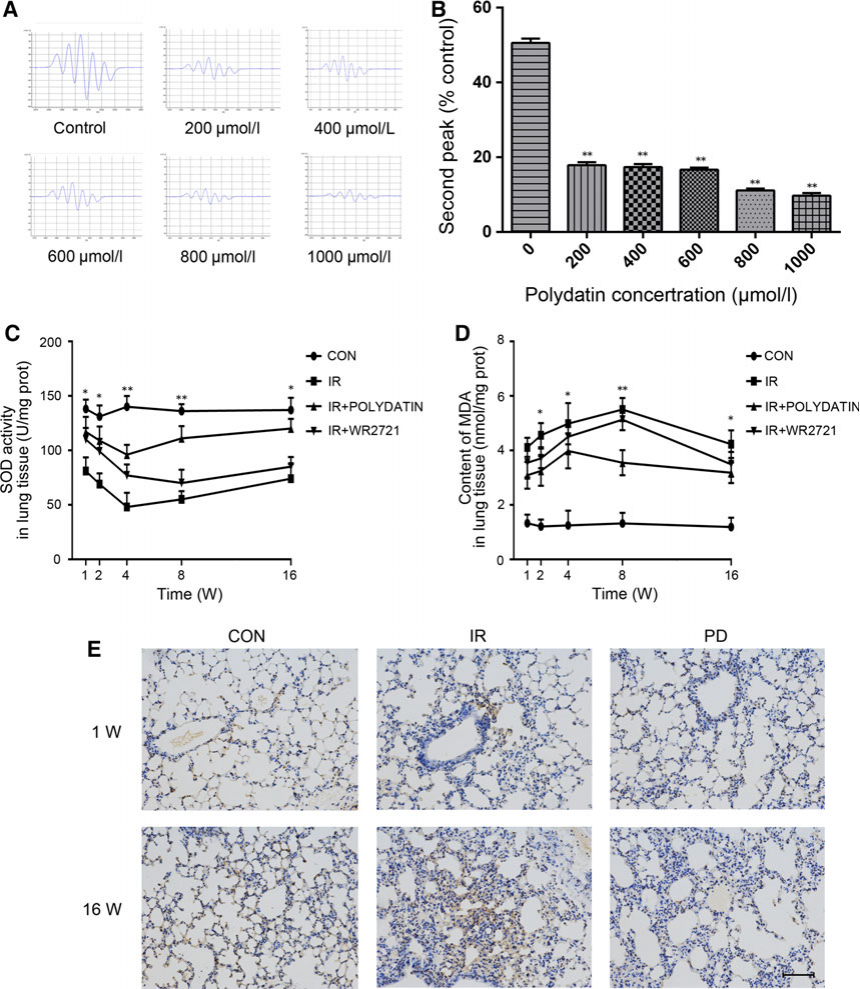
Polydatin scavenged free radicals and exerted an anti‐oxidative capacity. (**A**) Representative images of free radical signals from groups treated with different concentration of PD. (**B**) quantification analysis of second peak of free radicals in different groups. (**C**,** D**) The concentration of SOD and MDA in lung homogenate from mice with different treatments. (**E**) Representative images of TUNEL staining of lung tissues at 1‐ and 18‐week post‐irradiation. Values are given as mean ± SEM (*n* = 10), *P < 0.05 and **P < 0.01 *versus* single radiation group.

**Figure 8:**
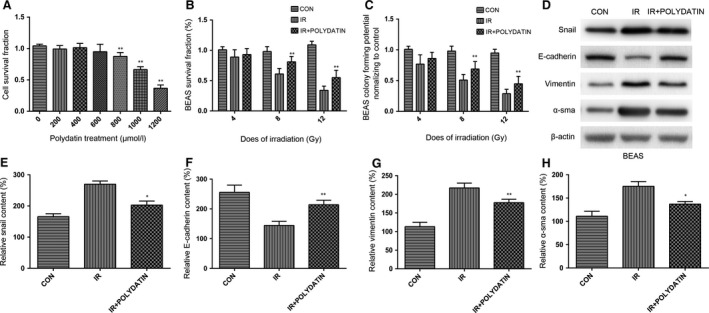
Polydatin protected normal alveolar epithelial cells from radiation induced cell death and EMT. (**A**) A bar graph of cell survival after treatments with different concentration PD. (**B**,** C**) Cell viability from different groups assayed by CCK8 assay and clone formation assay. (**D**) Western blot analysis of EMT markers in BEAS‐2B cells with/without PD treatment. (**E**–**H**) Quantification analysis of raw density of Western blot figures after in different groups. Values are given as mean ± SEM (*n* = 10), *P < 0.05 and **P < 0.01 *versus* single radiation group.

Free radicals were generated using a cell‐free Fenton reaction system. Our data showed that polydatin reduced the signal of free radicals in a dose dependent manner (Fig. 7A and B). In lung tissue homogenate, polydatin treatment significantly inhibited the decrease of SOD induced by irradiation (Fig. 7C). Compared with single irradiation group, polydatin treatment reduced the level of MDA in lung tissue homogenate (Fig. 7D). Polydatin treatment also significantly reduced the radiation‐induced apoptosis in alveolar epithelial cells and vascular endothelial cells (Fig. 7E). In CCK‐8 and clone formation assays, no toxicity was found in polydatin at the concentration less than 600 μM (Fig. 8A). Polydatin treatment significantly increased cell survival in both CCK‐8 and clone formation assays, showing a significant effect of radiation protection (Fig. 8B and C).

### Activation of Sirt3 might accounts for the radioprotective effects of Polydatin

**Figure 9:**
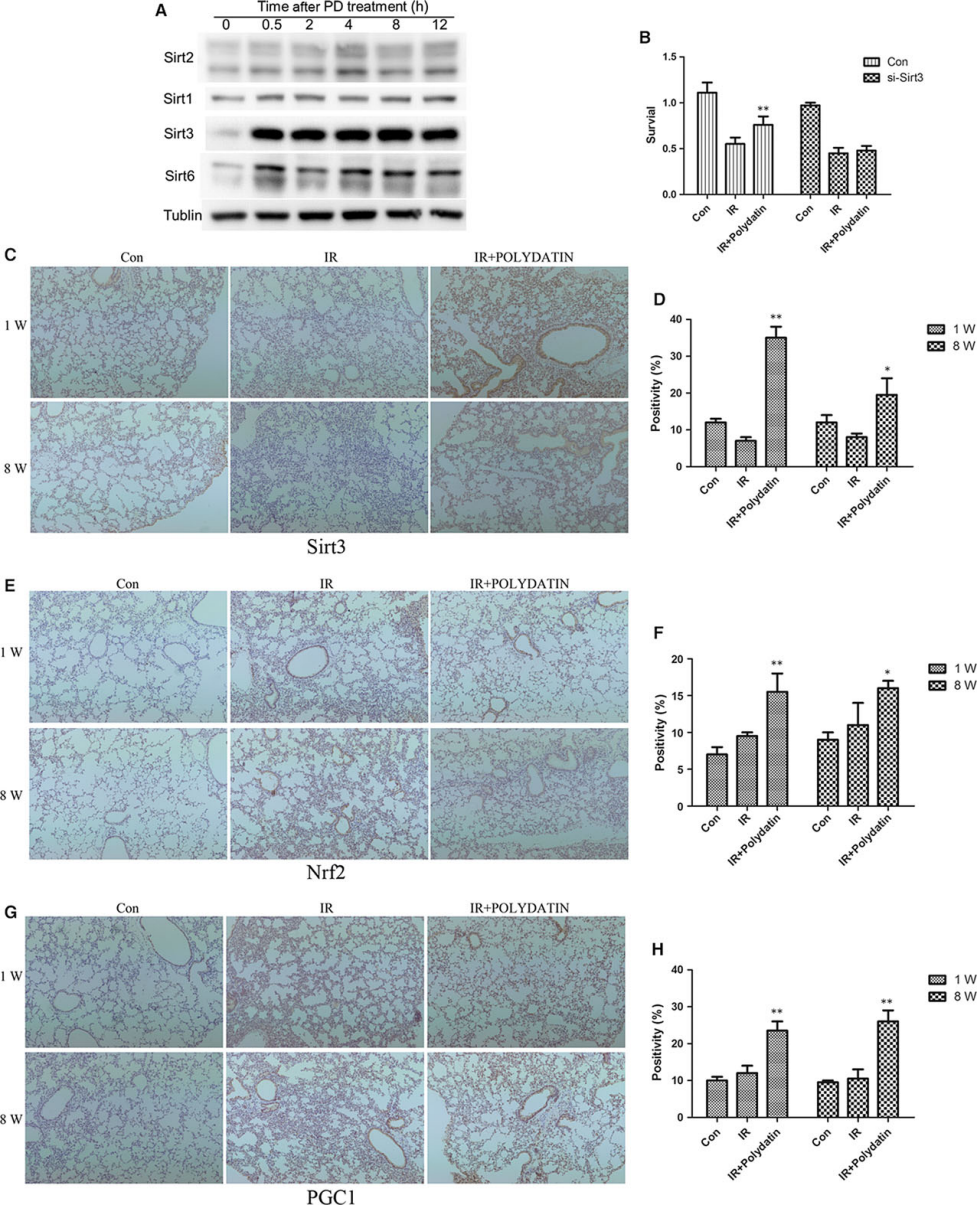
Polydatin elevated the level of Sirt3 and its related genes Nrf2, PGC1α. (**A**) Western blot analysis of Sirt family members including, Sirt1, Sirt2, Sirt3 and Sirt6. (**B**) Cell survival assay of cells transfected with Sirt3 siRNA and negative control after different treatments. (**C**,** E**,** G**) Representative images of Sirt3, Nrf2 and PGC1α IHC staining in lung tissues at 1 and 8 week after irradiation. (**D**,** F**,** H**) Bar graphs showing quantification analysis of Sirt3, Nrf2 and PGC1α‐positive cells in lung tissues from different groups. Values are given as mean ± SEM (*n* = 10), *P < 0.05 and **P < 0.01 *versus* single radiation group.

**Figure 10:**
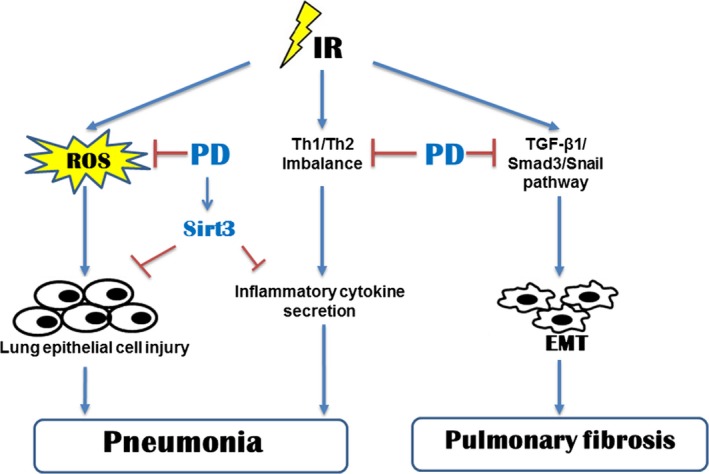
Modelling of PD in radiation‐induced lung injury. Irradiation of lung tissues induces pneumonitis and later chronic pulmonary fibrosis. PD alleviated acute pneumonitis through activating of Sirt3 and scavenging free radicals. PD also suppressed inflammatory cytokines and reversed Th1/Th2 imbalance. Pulmonary fibrosis was also inhibited by PD through inhibiting of EMT.

It has been reported that polydatin is a potent activator of Sirt1. Thus, our present study focused the influence of polydatin on Sirt family members, among which Sirt1, Sirt2 and Sirt6 were proved to be related with cellular radiosensitivity. In this study, we found that the level of Sirt3 was hugely elevated as early as 0.5 hr after polydatin treatment. And the increased ratio was much more than that of either Sirt1 or Sirt6 (Fig. 9A), suggesting Sirt3 as a potential target of polydatin. Using a Sirt3 siRNA, we found that the radioprotective effect of polydatin was abrogated in Sirt3 knock‐down cells (Fig. 9B). Then, we examined the influence of polydatin on Sirt3 and its related genes *in vivo*. In our RILI model, Sirt3 level also increased in polydatin‐treated groups in both 1‐ and 8‐week post‐irradiation (Fig. 9C and D), compared to single radiation groups. Nrf2 and PGC‐1α are upstream regulators of Sirt3, and we tested the expression of these two genes in RILI model. It was found that polydatin treatment significantly increased positive cells of Nrf2 (Fig. 9E and F) and PGC‐1α (Fig. 9G and H) staining. These data indicated that Sirt3 might be a novel potential target for polydatin, which provide information for its underlying mechanism (Fig. 10).

## Discussion

Radiation‐induced lung injury (RILI) is one of the most common complications in the radiotherapy of chest tumour as well as total body irradiation. It is a progressive and fatal disease which could cause severe impairments for respiratory system. Till now, there is no available treatment strategy for RILI. And the underlying mechanism is even unclear. In our study, we found that polydatin (PD) effectively mitigated the acute inflammation and lung fibrosis in late periods of RILI. PD also suppressed upregulation of TGFβ‐Smad3 signalling pathway and epithelial–mesenchymal transition, which was reported playing critical roles in lung fibrosis 22. Irradiation of lung tissues also caused a dysregulation of cytokines, exhibiting upregulation of inflammatory cytokines and shift of Th1/Th2 immunity 23. Our data showed that PD inhibited radiation‐induced inflammation and Th1/Th2 imbalance. In a cell‐free system, we also proved that PD effectively scavenged free radicals. We also found that PD induced several member of Sirt family, especially Sirt3, which contributed to its radioprotective effects.

Epithelial–mesenchymal transition (EMT) of epithelial cells is thought to play critical roles in pneumonitis fibrosis 24. It is characterized with a downregulation of E‐cadherin, and increases of Vimentin and α‐SMA. It was also reported that radiation could induce EMT in multiple tissues 25, 26, 27. In this study, we found PD treatment inhibited the upregulation of Vimentin and α‐SMA, and retained the level of E‐cadherin, indicating that PD inhibited EMT process in the late‐phase of RILI. We also used an *in vitro* model to detect the influence of PD on EMT process. It was found that PD inhibited changes of EMT markers of normal bronchial epithelial BEAS‐2B cells after irradiation. Previous studies have also revealed that TGF‐β‐Smad3 signalling pathway play important roles in radiation‐induced EMT process 28, 29, 30. TGF‐β recognizes and binds with its receptor TGFBR2 and ALK5, triggers the phosphorylation of Smad2/3. After binding with Smad4, Smad2/3 translocate into nucleus, inducing the encoding of collagen 10. Accumulating evidence also supports a role for TGF‐β and smad signalling in the development and progression of renal and lung fibrosis 9, 31. We demonstrated PD treatment significantly reduced the level of TGF‐β1, Smad3 and snail, indicating that PD might mitigate RILI through TGF‐β1‐Smad3 signalling pathway. We also observed that PD inhibited the level of TGF‐β1 and ET‐1 in serum of irradiated mice.

Ionizing radiation causes a lasting Th2 immune response, leading to imbalance of Th1 and Th2 immunity 32. Shift from Th1 to Th2 immune response is also involved in RILI 33. It has been reported that Th2‐related cytokines, IL‐4 and IL‐13 exerted distinct roles in tissue remodelling and fibrosis 34. IL‐4 and IL‐13 can also function separately or synergistic with TGF‐β1 to stimulate the deposit of collagen 11. While Th1‐related cytokine IFNγ was shown to play anti‐fibrosis and anti‐inflammation roles 35. Imbalance of Th1 and Th2 response also affects the immunity and leads to immunosuppression 11. Our data showed that PD reduced the level of IL‐4 and IL‐13, while retained the concentration of IFNγ, which was down‐regulated by irradiation. These data suggests that PD treatment reversed radiation‐induced imbalance of Th1 and Th2 response. In addition, PD also inhibited the level of inflammatory cytokine TNF‐α and PGE2, which was also reported to contribute to RILI 36.

About 60–70% radiation damage was attributed to the production of free radicals. Especially in RILI, the role of free radicals and reactive oxygen species (ROS) is very important. Briefly, there are three sources of ROS production. Firstly, the radiolysis of water produces multiple free radicals including ^·^OH, ^·^H and *e*_aq_. Secondly, inflammatory cells that were recruited to the lung produce ROS. Thirdly, ROS level increased from the leakage of electron transport chain of mitochondria 37. It was also found that PD attenuated H2O2 caused oxidative stress, showing that PD exerts anti‐oxidant roles 38. In our present study, we used a cell‐free system and found that PD scavenged free radicals directly. These data showed that the anti‐oxidative property of PD might account in part for the underlying mechanism of its protective effect on RILI in the acute phase. Silent information regulator (SIR) genes (Sirtuins) were found to exert key roles in cellular stress and are associated with ageing‐related diseases. And it has been proved that polydatin was a potent activator of Sirt1 in small intestine injury during haemorrhagic shock. Knockout of Sirt1 also sensitized glioma CD133‐positive cells to ionizing radiation. And we checked the expression of all Sirtuin members in response to polydatin treatment. Our data proved that after polydatin treatment, Sirt3, Sirt6 and Sirt1 were up‐regulated, and polydatin induced a huge increase in Sirt3. And knock‐down of Sirt3 abrogated the radioprotective effects of polydatin in normal alveolar epithelial cells. Polydatin treatment also increased the Nrf2 and PGC1‐positive cells in RILI model. Our results suggest that Sirt3 might be a novel potential target for polydatin.

In conclusion, our data showed that PD‐mitigated inflammation and the fibrosis in later phase of lung tissues in RILI model. The mechanism might be related to the inhibition of EMT process and the scavenging of free radicals. PD also activated Sirt3 and inhibited inflammatory cytokines. Our data suggests PD as a potential radioprotector in the clinical treatment of RILI.

## Conflict of interest

The authors have no conflicts of interest to disclose.
